# Ontology-Driven and Human-Centric Digital Twins in Hospitality: A Survey and Research Agenda

**DOI:** 10.3390/s26092764

**Published:** 2026-04-29

**Authors:** Desiree Manzano-Farray, Moises Segura-Cedres, Carmen Lidia Aguiar-Castillo, Victor Guerra-Yanez, Rafael Perez-Jimenez

**Affiliations:** 1 Instituto para el Desarrollo Tecnológico y la Innovación en Comunicaciones (IDeTIC), Universidad de Las Palmas de Gran Canaria (ULPGC), Juan de Quesada 30, 35001 Las Palmas de Gran Canaria, Spain; desiree.manzano@ulpgc.es (D.M.-F.); moises.segura101@alu.ulpgc.es (M.S.-C.); rafael.perez@ulpgc.es (R.P.-J.); 2 Escuela Superior de Ingeniería y Tecnología, Universidad Internacional de La Rioja (UNIR), Avda. de la Paz, 137, 26006 Logroño, Spain; victor.guerra@unir.net

**Keywords:** digital twins, social digital twins, human-centric digital twins, tourism, hospitality, smart tourism, ontology-driven systems, semantic interoperability, human-in-the-loop, systematic literature review

## Abstract

**Highlights:**

**What are the main findings?**

- Digital Twin research in tourism and hospitality is predominantly infrastructure-focused, with limited development of human-centered models.
- Current implementations show high technological heterogeneity but lack ontology-driven models, semantic integration, governance considerations, and real-time synchronization.

**What are the implications of the main findings?**

- Advancing towards Social Digital Twins requires integrating human actors, social interactions, and governance within unified modelling frameworks.
- Semantic and ontology-driven approaches are essential to enable interoperability, explainability, and human-centric decision-making in service environments.

**Abstract:**

Digital Twins (DTs) are increasingly explored in tourism and hospitality as enabling technologies for smart destinations, service optimization, and data-driven decision-making. Yet these environments are inherently human-centered. Existing DT implementations, however, are largely technology-driven and focus mostly on infrastructures and operational processes. This study presents a systematic literature review of DT applications in tourism and hospitality. It combines a comparative taxonomy with a technological and data-oriented analysis to examine how these systems are currently conceptualized, implemented, and integrated. The review analyzes 42 studies, classifying them by application level, twin focus, architectural approach, and human integration. The results show a strong dominance of destination- and facility-level DTs, limited human-centered models, and a prevalent use of varied sensing technologies. There is limited attention to interoperability and semantic integration. Governance, socio-technical aspects, and real-time synchronization mechanisms are also mostly underexplored. Based on these findings, this study identifies key research gaps and calls for a shift towards Social Digital Twins (SDTs). SDTs integrate human actors, social interactions, and governance within unified modelling frameworks. This transition will require advances in semantic and ontology-driven architectures. Greater attention to privacy, trust, and user acceptance in data-intensive service environments is also needed.

## 1. Introduction

Digital Twin (DT) concept has evolved from its origins in industrial engineering and cyber–physical systems into a key paradigm for modelling, monitoring, and optimizing complex environments through the integration of physical and digital systems [1,2,3]. Advances in sensing technologies, Internet of Things (IoT) infrastructures, artificial intelligence, and data analytics have enabled DTs to move beyond asset-level representations towards more interconnected and data-driven systems capable of supporting real-time decision-making across multiple domains [4,5,6,7].

In recent years, this paradigm has been progressively adopted in service-oriented sectors, including tourism and hospitality, where DTs are increasingly used to support smart destination management, infrastructure monitoring, and service optimization [8,9]. However, unlike industrial environments, tourism and hospitality systems are inherently socio-technical, characterized by complex interactions between technological infrastructures, organizational processes, and human actors such as employees and customers [10,11]. In these contexts, value is co-created through service interactions and contextual decision-making, which cannot be fully captured by infrastructure-centric or purely data-driven DT approaches.

Despite the growing interest in DT applications within tourism and hospitality, existing research remains fragmented and largely focused on technological implementations, with limited attention to human integration, governance mechanisms, and the broader socio-technical dynamics of service systems. Moreover, the increasing diversity of sensing technologies and data sources introduces additional challenges related to interoperability and data integration, underscoring the need for more structured semantically grounded approaches to DT development [12]. At the same time, recent advances in ontology-driven architectures and human-centric AI suggest new opportunities to move towards more comprehensive and socially aware DT systems capable of representing not only physical infrastructures but also human behavior, interactions, and organizational contexts [12,13,14,15,16].

In this context, this study aims to systematically analyze the current state of research on DTs in tourism and hospitality and to identify the main patterns, limitations, and future research directions in the field. Specifically, the review addresses four research questions related to (i) the application of DTs in tourism and hospitality, (ii) the technological and architectural approaches used, (iii) the integration of human actors within DT systems, and (iv) the identification of research gaps and future research directions. By combining a comparative taxonomy with a technologic and data-oriented analysis, this study provides a structured framework for examining how DT systems are currently conceptualized and implemented in service environments.

Building on this analysis, the paper also advances the concept of Social Digital Twins (SDTs) as an evolution of traditional DT architectures. SDTs extend conventional approaches by integrating human actors, social interactions, and governance dimensions within a unified modelling framework, enabling a more comprehensive representation of the socio-technical complexity that characterizes tourism and hospitality systems.

In doing so, this study departs from predominantly technology-driven perspectives in the existing literature by offering a comprehensive and structured analysis of DT systems that explicitly accounts for the socio-technical complexity of tourism and hospitality environments.

The remainder of this paper is organized as follows. Section 2 presents the conceptual and contextual foundations of DTs and their evolution towards human-centric and social paradigms. Section 3 describes the methodology adopted for the systematic literature review. Section 4 introduces the conceptual framework for classifying DT applications in tourism and hospitality. Section 5 examines the role of human-centric and social DTs, while Section 6 discusses the main socio-technical challenges associated with their adoption. Section 7 presents a comparative analysis, a taxonomy, and an identification of research gaps. Finally, Section 8 summarizes the main conclusions and outlines future research directions.

## 2. Background and Contextual Foundations

The concept of the DT originated in industrial engineering and cyber–physical systems as a digital representation of a physical asset connected through continuous data exchange. Early definitions describe DTs as virtual counterparts that can replicate the structure, state, and behavior of physical systems throughout their lifecycles [1,17]. These conceptualizations converge around three core elements: a physical entity, its digital representation, and a bidirectional data connection enabling monitoring, simulation, and optimization.

Initial DT applications were primarily developed in industrial and manufacturing contexts, where they were used to monitor assets, predict failures, and optimize operational processes [18]. With the expansion of IoT infrastructure, cloud computing, and advanced analytics, DTs have evolved into integrated cyber–physical systems capable of modeling entire production environments and supporting system-level decision-making [3,4,19,20]. This evolution reflects a shift from asset-level monitoring toward more complex, interconnected representations of operational systems [7].

Although the DT paradigm matured and its application extended beyond industrial domains to areas such as smart cities, healthcare, transportation, and service systems [12], most early applications remained focused on physical infrastructure and engineering systems. However, in service-oriented environments such as tourism and hospitality, this infrastructure-centric perspective is insufficient. These contexts operate as socio-technical systems in which performance emerges from interactions between employees, customers, organizational processes, and technological infrastructures [8,10]. Consequently, the DT architecture must evolve to represent not only physical assets but also service workflows, human activities, and the contextual conditions that shape operational outcomes. The increasing complexity of the service environment introduces significant challenges in data integration and interoperability. Tourism organizations typically rely on heterogeneous information systems, including property management systems, reservation platforms, IoT infrastructures, and customer interaction channels, which generate diverse and distributed data streams [9]. In this context, semantic modelling approaches based on ontologies provide mechanisms for structuring domain knowledge and enabling interoperability across systems [13,21]. Previous research has proposed an ontology-driven DT framework for hotel front-desk operations that integrates IoT devices, reservation platforms, and service workflows within a semantic model capable of supporting predictive analytics and operational optimization [15] and has developed systems to capture physiological signals and spatial events in real time, enabling adaptive workload monitoring and improved situational awareness in front-desk operations [16]. By combining multimodal sensing technologies with semantic data models, these architectures provide a more comprehensive representation of service environments.

Building on this perspective, there is a need for human-centric DT architectures capable of capturing behavioral, contextual, and organizational dimensions of service systems. Human-Centric AI shifts the focus from purely technical optimization toward systems that support human decision-making, preserve agency, and ensure transparency, fairness, and accountability [22]. In service environments, operational decisions are rarely purely technical. Queue management, workload distribution, and resource allocation directly affect employee well-being and customer experience. Consequently, AI systems must be interpretable, support human-in-the-loop decision-making, and align with organizational norms and ethical constraints [23]. Human-centric design emphasizes explainability, data minimization, privacy-aware sensing, and governance mechanisms that prevent function creep and excessive surveillance. These aspects are particularly relevant when wearable devices, indoor localization, or behavioral analytics are deployed in workplaces.

From a socio-technical perspective, DTs must therefore incorporate roles, interactions, and organizational constraints as integral components of the system [24]. In this context, the ontology-oriented SDT can therefore be understood as a layered socio-technical architecture in which semantic models represent not only operational entities—such as queues, zones, and service tasks—but also social constructs including roles, interaction episodes, perceived fairness, and governance constraints [25]. In contrast with traditional DTs centered on physical assets, the SDT explicitly addresses environments where human interaction is the primary driver of performance and where organizational acceptance and trust are essential for deployment [26]. By combining heterogeneous data integration, semantic knowledge models, and human-centric design principles, SDTs enable the modeling of complex service environments where operational performance depends on interactions between technological systems and human behavior [25,26,27].

**Figure 1:**
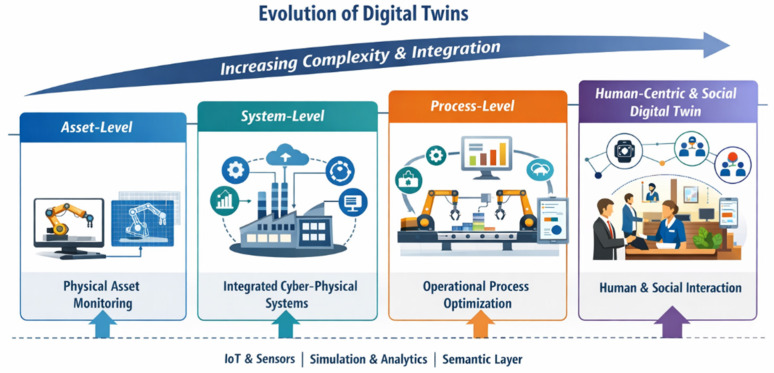
Evolution of DTs toward human-centric systems. The figure illustrates the progressive increase in system complexity and integration, from asset-level DTs focused on physical monitoring to system- and process-level representations, and finally to human-centric and social DTs that incorporate human actors, interactions, and socio-technical dynamics within the modeled environment. The different stages depicted in the figure reflect this transition and highlight the increasing role of human and organizational dimensions in DT development.

This evolution from asset-level monitoring to human-centric and socially aware DT architectures is summarized in Figure 1.

## 3. Methodology of the Review

This study aims to systematically analyze the current state of research on DTs in tourism and hospitality contexts. The review is guided by four research questions addressing: (i) the application of DTs in tourism and hospitality, (ii) the technological and architectural approaches used, (iii) the integration of human actors within DT systems, and (iv) the identification of research gaps and future research directions.

The literature search was conducted in two major scientific databases: Web of Science and Scopus [28,29]. These databases were selected due to their broad coverage of peer-reviewed publications across engineering, information systems, and tourism-related research. The search was performed between December 2025 and March 2026 using and including all publications indexed up to that date. The search string was designed to capture both core DT concepts and their application within tourism and hospitality contexts:

(“digital twin*” OR “digital shadow*” OR “digital model*” OR “cyber-physical system*” OR “virtual replica*”)

AND

(“tourism” OR “hospitality” OR “hotel*” OR “destination*” OR “service system*” OR “guest experience”)

The query was applied to title, abstract, and keywords fields. To enhance coverage, a snowballing strategy (backward and forward citation tracking) was applied to the initially selected papers. As seen, the search strategy combined terms related to DT technologies with keywords associated with tourism and hospitality contexts (e.g., tourism, hospitality, hotel, smart tourism, and service industry), and was applied to titles, abstracts, and keywords. Results were limited to peer-reviewed journal articles, conference papers, and review papers. Additionally, only studies published in English were included in the review. No temporal restrictions were applied in order to capture the full evolution of the field and assess its development over time. The earliest records retrieved date from 2017 (WoS) and 2018 (Scopus), confirming that research on DTs in tourism and hospitality is a relatively recent phenomenon.

The initial search yielded 157 records in Scopus and 234 in WoS, totaling 391 publications. After removing 53 duplicates via DOI comparison and manual verification, 338 records were retained for screening. A relevance assessment based on titles, abstracts, and keywords was then conducted to determine the suitability of each study. Articles were classified as (i) relevant when they explicitly addressed DT technologies in tourism, hospitality, or related service contexts; (ii) contextual when they discussed related digital technologies without focusing on DT implementations; (iii) excluded when they were not related to DT technologies or were situated in domains outside the scope of this review. This classification was designed to ensure conceptual consistency and analytical comparability across the selected studies. In particular, only studies classified as relevant were included in the in-depth analysis, as they provided sufficient detail on DT implementations to be systematically examined using the proposed taxonomy. Contextual studies, although valuable for understanding the broader discourse, were excluded from the comparative analysis due to their lack of explicit DT modelling or implementation characteristics.

A significant number of exclusions (273 studies) resulted from the ambiguity of the term “service industry,” which is frequently used in engineering literature to refer to industrial service systems rather than tourism or hospitality contexts. Additionally, several studies mentioned DTs in keywords but did not develop, analyze, or reference DT concepts in the abstract, leading to their exclusion. This screening process resulted in 36 studies classified as relevant and 29 as contextual. To complement the database search, a snowballing procedure was applied to the relevant studies, including both backward (reference analysis) and forward (citation tracking) searches. This process identified 42 additional studies, which were evaluated using the same screening criteria. As a result, 10 additional studies were classified as relevant and 32 as contextual.

**Figure 2:**
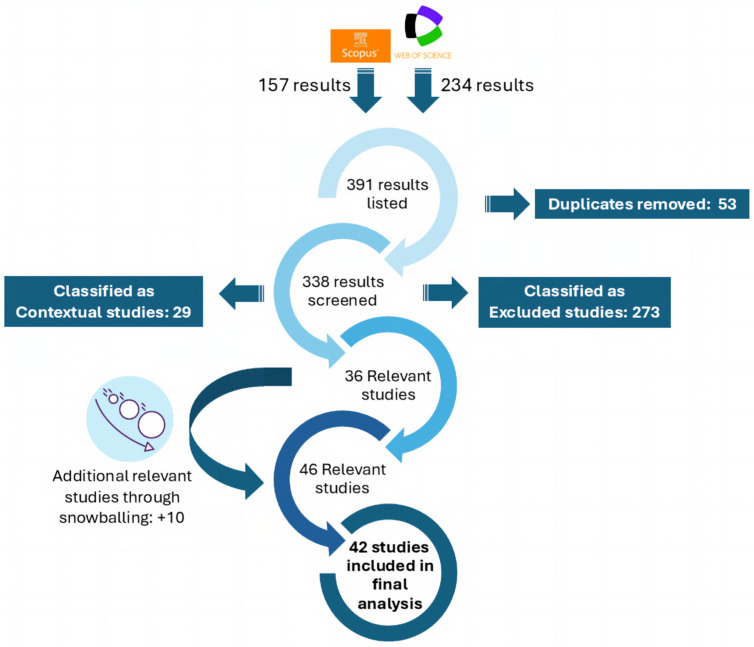
Flow diagram illustrating the identification, screening, and selection process of the studies included in the systematic literature review. The figure presents the number of records retrieved from Scopus and Web of Science, the removal of duplicates, the screening and classification of studies, and the incorporation of additional records through snowballing, leading to the final set of studies analyzed.

The final dataset consisted of 46 relevant studies. Full-text access was obtained for 42 of these publications, which were subsequently analyzed in depth, while four studies were excluded from the analytical stage due to access limitations. The selected studies were examined using a structured data extraction framework designed to support comparative analysis. For each article, key variables were recorded, including application level, twin focus, architectural approach, and degree of human integration. Additional aspects, such as the technologies used, data-acquisition mechanisms, and real-time synchronization, were also considered to assess the technological maturity of the proposed DT systems and to identify potential limitations. The study selection process is summarized in Figure 2, which illustrates the identification, screening, and selection of studies [30].

## 4. Conceptual Framework for the Classification of Digital Twins in Tourism and Hospitality

The growing body of research on DTs in tourism and hospitality reveals technological diversity but also clear patterns of concentration in specific application domains and modeling approaches. Existing studies tend to focus predominantly on destination-level applications, environment-centric representations, and limited forms of human integration, treating individuals mainly as system users or data sources. This relative homogeneity across key dimensions contrasts with the diversity observed in technological implementations and data sources, highlighting important gaps in the current development of DT systems in service-oriented environments.

To systematically capture these patterns and identify the main limitations of existing approaches, this study proposes a conceptual framework to classify DT applications in tourism and hospitality contexts, designed to describe, structure, and synthesize the current body of knowledge. The framework is structured around four analytical dimensions—application level, twin focus, architectural approach, and human integration—which together reflect the multi-level, socio-technical, and data-intensive nature of DT systems [4,7,24].

**Figure 3:**
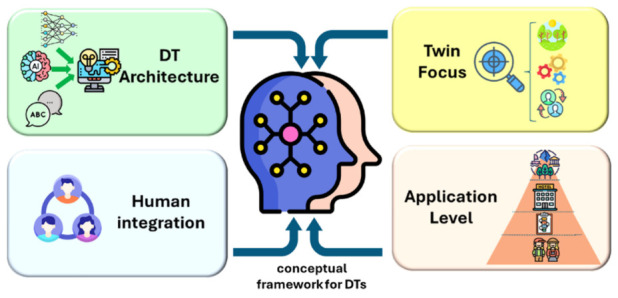
Conceptual framework for DTs in tourism and hospitality. The framework organizes DT systems according to four main dimensions (application level, twin focus, architectural approach, and human integration) providing a structured perspective to analyze their design and implementation in service-oriented environments.

The application level refers to the scope at which the DT operates. DTs may be developed at the destination level, representing large-scale tourism ecosystems; at the facility or organizational level, focusing on infrastructure such as hotels or heritage sites; at the process level, modeling operational workflows; or at the individual level, representing specific users or actors within the system. This distinction reflects the multi-level nature of DT applications in complex systems [7]. The twin focus describes the DT’s primary modeling perspective. Existing approaches may emphasize environmental conditions, physical assets, operational processes, or human actors and interactions, while some systems adopt hybrid configurations. This distinction is consistent with the evolution of DTs from asset-centric models toward more process-oriented and human-centric representations in complex systems [3,4,7,19,20]. Architectural approach refers to the technological paradigm underlying the DT implementation. These include data-driven approaches, simulation-based models, ontology-driven architectures enabling semantic integration, AI-enhanced systems incorporating advanced analytics, and hybrid configurations combining multiple approaches. This classification demonstrates the evolution toward more complex and interconnected DT architectures [3,4,19,20]. Finally, human integration captures the role assigned to human actors within the DT. Prior research has shown that many DT systems represent humans as system users or data sources, while more advanced approaches incorporate human actors as integral components of the system, enabling more human-centric and socio-technical representations [15,16,24]. It is important to note that these categories are not mutually exclusive. In practical implementations, a single actor may simultaneously occupy multiple roles within the system, for instance as a system user, a sensed entity, and a core model component. Accordingly, studies were classified into all applicable categories when multiple forms of human integration were identified. Taken together, these dimensions provide a structured way to capture both the dominant patterns and the underexplored areas in current DT research in tourism and hospitality. By combining system scope, modelling perspective, technological design, and human integration, the proposed framework reflects the multi-level and socio-technical nature of DT systems in service environments. As illustrated in Figure 3, this framework organizes DT applications across four complementary dimensions, highlighting their relationships and their roles in shaping current research approaches. On this basis, Section 7 applies the framework to the 42 reviewed studies to develop a comparative analysis and taxonomy of DT applications in tourism and hospitality.

## 5. Human-Centric and Social Digital Twins

The evolution of DTs from asset-oriented monitoring systems toward complex cyber–physical representations has increased interest in human-centric and social DT paradigms. While early implementations focused on predictive maintenance and industrial optimization [1,2,4,5,6], service-oriented domains such as hospitality depend on human behavior, organizational processes, and contextual interactions [8,10,11]. In these environments, value is co-created through interactions between employees, customers, and service infrastructures, with frontline employees playing a key role in shaping service experiences [31] and digital technologies increasingly influencing service delivery processes [32].

The results of this systematic review highlight the limitations of current DT implementations in capturing this socio-technical complexity. Although 36 of the 42 studies analyzed incorporate some form of human integration, humans are typically represented in peripheral roles. In many cases, they appear mainly as users interacting with visualization or immersive interfaces [33,34,35,36], while in others they are included indirectly through sensing or tracking technologies, where behavioral data are used as contextual inputs rather than explicitly modeled elements [37,38,39,40]. Notably, only two studies explicitly represent humans as core entities within the DT itself [15,16]. These findings reveal a clear gap between the socio-technical nature of tourism and hospitality environments and current DT implementations, where human actors remain largely peripheral rather than central modelling elements.

In this context, semantic modelling and ontology-driven DTs offer promising approaches for representing the complex relationships between actors, processes, and resources in tourism systems. Ontologies enable the formal representation of entities and relationships, allowing heterogeneous data sources to be integrated within a shared semantic framework [12,13,14]. By introducing a semantic layer that links physical entities, digital systems, and operational processes, ontology-driven DTs facilitate contextual data interpretation and support advanced reasoning. Empirical studies illustrate this potential in hospitality contexts, where ontology-based DT architectures integrate operational workflows, guest interactions, and sensing technologies to enable real-time analysis and decision support [15,16,41]. Semantic technologies play a key role in enabling this integration by providing a shared conceptual layer that allows heterogeneous data to be interpreted consistently across systems. Through ontology-based modeling, entities such as guests, staff members, rooms, services, and devices can be represented within a unified semantic structure that preserves contextual relationships and operational meaning [15,16]. This semantic layer reduces ambiguity when combining information from independent systems and supports machine-interpretable representations of complex service environments. When integrated into DT platforms, semantic models also enable reasoning and knowledge inference enhancing decision support. By analyzing relationships between entities and contextual variables, DT systems can derive operational insights from existing data. For example, reservation data indicating an imminent surge in guest arrivals may allow the system to anticipate increased reception workload and recommend adaptive task-allocation strategies. Similarly, patterns detected in service requests or environmental conditions may reveal operational anomalies or service bottlenecks. These capabilities allow ontology-driven DTs to evolve from passive monitoring systems into context-aware decision-support platforms capable of interpreting complex operational dynamics [3].

Despite these advantages, implementing human-centric and socially aware DTs presents several challenges, including integrating heterogeneous data sources, scaling semantic models and reasoning processes, and addressing organizational and governance issues related to privacy, transparency, and user acceptance. Overall, human-centric and social DTs represent an important evolution of the DT paradigm. By combining heterogeneous data integration, semantic knowledge models, and reasoning capabilities, these systems enable more context-aware and adaptive service management. In this context, recent ontology-driven approaches in hospitality increasingly adopt structured architectural perspectives, organizing Digital Twin systems into interconnected layers that integrate data acquisition, data management, semantic modelling, analytics, and human interaction components [15,16,41]. These layered frameworks facilitate interoperability across heterogeneous systems and enable the combination of real-time data processing with semantic reasoning and decision support. At the same time, such architectures are embedded within broader socio-technical ecosystems, where governance mechanisms, regulatory requirements, and stakeholder interactions play a central role in shaping system design and adoption.

## 6. Socio-Technical Challenges and Adoption of Digital Twins in Hospitality and Tourism

DTs in hospitality and smart destination environments operate within complex socio-technical systems in which sensing technologies, artificial intelligence, organizational structures, and human actors are tightly interconnected. These systems rely on data collected through infrastructure sensors, wearable devices, indoor positioning systems, and computer vision technologies to support real-time monitoring and operational decision-making. While these capabilities enable more adaptive and data-driven service management, they also introduce significant privacy, ethical, and governance challenges, particularly in workplace environments where employees may be continuously monitored [42]. In such contexts, data protection and responsible data use become central design requirements rather than secondary considerations. These challenges also point to the need for regulatory and policy frameworks that can guide the responsible integration of data-driven technologies in tourism and hospitality environments. In the European context, regulatory frameworks such as the General Data Protection Regulation (GDPR) emphasize principles including data minimization, purpose limitation, and transparency, requiring organizations to collect only data that is strictly necessary and to ensure appropriate safeguards for personal information [43]. However, in sensor-rich environments, even aggregated or seemingly anonymous data, such as location traces or behavioral patterns, may enable indirect identification, reinforcing the need for privacy-by-design approaches that prioritize anonymization and controlled data use [44]. At the same time, transparency plays a critical role in shaping employee perceptions, as workers must understand how data are collected, processed, and used within DT systems. Without clear communication and governance mechanisms, these systems risk being perceived as surveillance tools, undermining trust and acceptance [45].

These challenges are closely linked to the broader need to embed Responsible and Trustworthy AI within DT architectures. In hospitality and smart destination environments, AI-driven DTs support decision-making across service operations, including workload allocation, queue management, and visitor flow optimization. Because these decisions directly affect employees and customers, ensuring fairness, explainability, robustness, and accountability becomes essential [46,47,48,49]. These principles extend beyond ethical guidelines and must be operationalized within system design to ensure legitimacy, compliance, and long-term adoption.

At the organizational level, the effective deployment of DTs depends on institutions’ readiness to manage these socio-technical systems. This includes not only technological infrastructure but also human capabilities, governance structures, and cultural alignment. Organizations must develop data-driven competencies, redesign workflows, and establish coordination mechanisms that allow different stakeholders—such as hotels, destination management organizations, and public authorities—to operate within interconnected DT ecosystems [50,51,52,53]. Without such alignment, the benefits of advanced DT systems may not translate into effective operational or strategic outcomes.

Within this socio-technical framework, human-in-the-loop (HITL) decision-making emerges as a key mechanism for balancing automation and human judgment. Rather than replacing human decision-makers, DT systems increasingly support hybrid decision processes in which humans monitor, validate, and intervene in algorithmic outputs [54,55,56]. Research on AI-enabled decision-making highlights that effective deployment of intelligent systems often requires combining automation with human judgment rather than fully replacing human decision makers. In this context, [57] describe an automation–augmentation paradox, arguing that automation and augmentation are not mutually exclusive approaches but interdependent mechanisms that must be balanced within organizational decision processes. Recent work on hybrid intelligence systems further emphasizes the importance of carefully designing the interaction between human and artificial agents. Rather than positioning humans or AI as the sole decision authority, emerging perspectives highlight the need for collaborative architectures in which decision control is dynamically shared between humans and intelligent systems depending on context, task complexity, and risk levels [58]. This approach reflects the need to combine analytical capabilities with contextual knowledge, particularly in service environments where decisions depend on situational awareness and social interaction [9]. Finally, the deployment of DTs in tourism and hospitality requires a holistic approach that integrates privacy protection, responsible AI, organizational readiness, and human-centered decision-making within a unified socio-technical framework. Addressing these interdependent dimensions is essential to ensure not only technological effectiveness but also social acceptance, regulatory compliance, and sustainable adoption.

## 7. Comparative Analysis and Taxonomy

**Figure 4:**
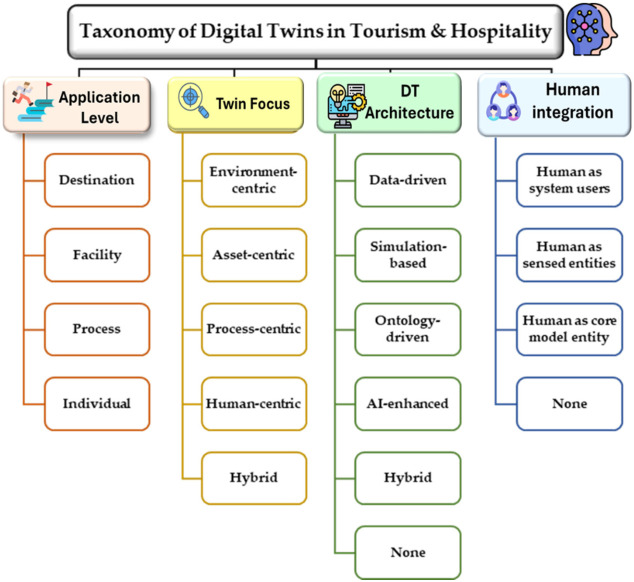
Classification framework of DT research in tourism and hospitality. The framework organizes the analyzed studies according to four main dimensions: application level, twin focus, architectural approach, and human integration. Each dimension includes multiple categories that reflect different system characteristics and design approaches.

To enable a structured comparison of the reviewed literature, this study adopts the classification framework introduced in Section 4, which organizes DT research in tourism and hospitality along four complementary dimensions: application level, twin focus, architectural approach, and human integration. Figure 4 summarizes this taxonomy, which was used to systematically classify the 42 analyzed studies. Rather than representing isolated categories, these dimensions capture different but interrelated aspects of DT systems, including the level at which they are implemented, the primary element being modelled, the underlying technological architecture, and the extent to which human actors are integrated into the system.

### 7.1. Comparative Table of Existing Works

To complement the proposed taxonomy, the reviewed studies were comparatively classified according to the four analytical dimensions defined in Section 4: application level, twin focus, architectural approach, and human integration. This classification enables a structured comparison of the 42 analyzed studies and facilitates the identification of recurring patterns in how DTs are conceptualized and implemented in tourism and hospitality contexts.

Table 1a–c presents the comparative classification of the reviewed studies. To improve clarity, studies sharing identical characteristics across all four dimensions are grouped within the same row. This structured comparison reveals consistent patterns across the literature regarding scale, modelling focus, technological configuration, and the treatment of the human element. The comparative classification is organized into three tables, grouping the analyzed studies according to their application level: destination (Table 1a), facility (Table 1b), and process (Table 1c). This grouping is introduced as a practical strategy to reduce visual complexity and enhance interpretability, given the sample size. Importantly, this organization does not imply a prioritization of the application-level dimension over the others. Instead, it reflects the relatively lower heterogeneity in this dimension, allowing clearer grouping of studies without affecting the analytical framework. The analysis remains grounded in the four dimensions defined in this study, which are considered jointly in interpreting the results.

Across the reviewed literature, DT applications are predominantly developed at the destination and facility levels. Destination-level implementations are typically associated with smart tourism destinations and urban-scale monitoring systems, while facility-level DTs focus on infrastructures such as hotels, museums, or heritage sites. In contrast, process-level applications are less common and are mainly used in operational monitoring and service management contexts. Notably, no studies were identified at the individual level, indicating that the representation of individual actors remains largely unexplored.

In terms of twin focus, most studies adopt environment-centric approaches, particularly in destination-level applications where DTs are used to monitor environmental conditions, spatial dynamics, or infrastructure status. Facility-level implementations more frequently incorporate asset-centric perspectives, reflecting the importance of infrastructure and equipment management. Process-centric approaches are also present, although less dominant. By contrast, human-centric DTs remain extremely limited, with only two studies placing human actors at the core of digital representation.

Regarding architectural approaches, literature is strongly dominated by data-driven architectures, typically based on sensor networks, real-time data platforms, and monitoring systems. Several studies adopt hybrid architectures combining multiple technological paradigms, while simulation-based and AI-enhanced approaches are less common. Ontology-driven DTs are also limited in number, despite their relevance for modelling complex socio-technical relationships. In addition, some contributions remain conceptual and do not specify a concrete technological architecture.

The analysis of human integration further highlights a consistent pattern across the reviewed studies. In most cases, human actors are incorporated in a limited manner, primarily as system users interacting with DT or as sensed entities whose data are used as inputs for monitoring and analysis. However, these representations rarely position humans as central modelling components within the system. Only two studies explicitly define human actors as core entities within the DT, reinforcing the limited development of human-centered approaches in the current literature.

**Figure 5:**
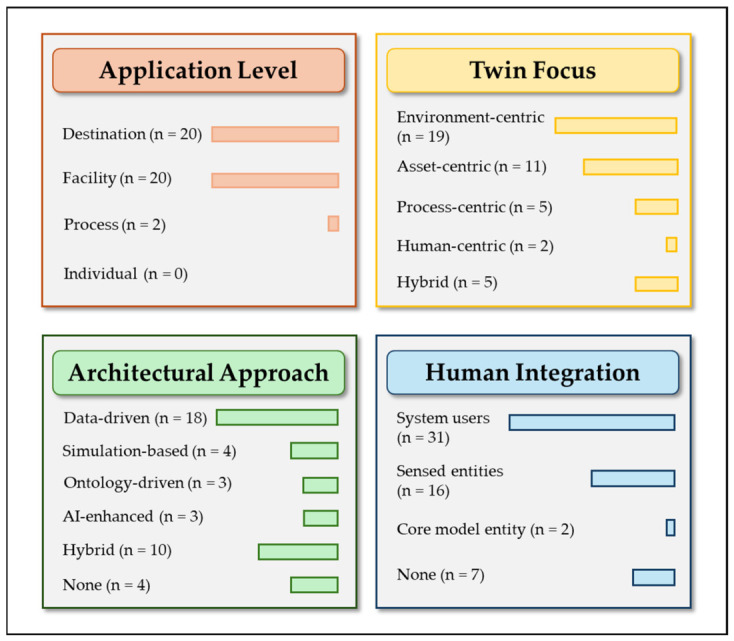
Distribution of the reviewed studies across the four dimensions of the proposed DT taxonomy. The figure presents the number of studies classified within each category of application level, twin focus, architectural approach, and human integration, highlighting the distribution of the sample across the different dimensions. Articles may contribute to more than one category when multiple forms of human integration are present.

To provide a clearer overview of how the reviewed studies are distributed across the proposed taxonomy, Figure 5 summarizes the empirical distribution of the analyzed articles across the four classification dimensions: application level, twin focus, architectural approach, and human integration. The figure visually highlights the concentration of studies in certain categories, particularly destination- and facility-level applications, environment-centric DTs, and data-driven architectures. With regard to human integration, the reviewed studies are predominantly associated with humans as system users, while a smaller number consider humans as sensed entities, and only a very limited number position them as core model entities. At the same time, the figure illustrates the limited presence of some categories, such as individual-level DTs and human-centric approaches, which appear only marginally in the current literature.

While the proposed taxonomy captures the structural dimensions of DT systems, an additional technological characteristic frequently discussed in the literature is the presence of real-time synchronization between the physical and digital systems. To complement the classification presented above, Table 2 summarizes the reviewed studies according to their level of real-time synchronization (yes, partial, or no).

The distribution of real-time synchronization across the reviewed studies highlights a certain degree of variability in how this feature is addressed in the literature. A total of 9 studies explicitly incorporate real-time synchronization mechanisms within their proposed systems, while 14 include this capability only partially. In contrast, 19 studies do not clearly specify the presence of real-time data exchange in their implementations, although in some cases it is mentioned as a potential extension or future development. These results illustrate the diversity of approaches in the operationalization of DT systems in tourism and hospitality contexts. In this regard, the presence and role of real-time synchronization appear to vary depending on the scope, maturity, and technological orientation of the proposed systems. This variability is further discussed in Section 7.4 in relation to the broader conceptualization of DTs.

### 7.2. Technological and Data Foundations of DT Systems

**Figure 6:**
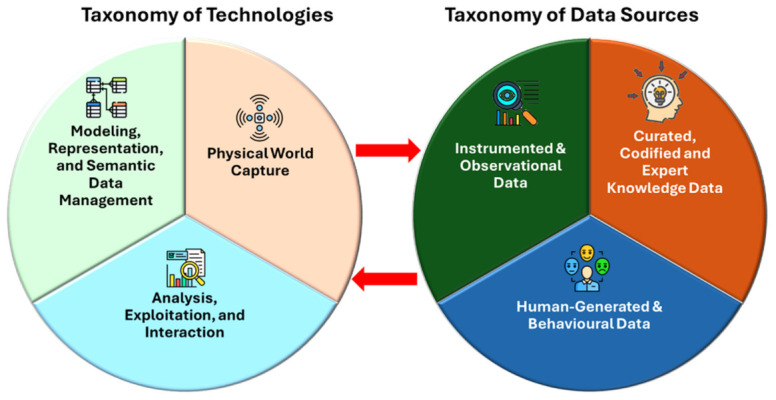
Technological and data foundations of DT systems in tourism and hospitality. The figure presents two complementary taxonomies: technologies, including physical world capture, analysis and interaction, and modeling and data management; and data sources, including instrumented and observational data, human-generated data, and curated knowledge.

To complement the comparative analysis presented previously, an additional examination of the technological components and data-acquisition mechanisms used in the reviewed studies was conducted. Table 3 and Table 4 synthesize the technological and data-related foundations of the studies analyzed in this survey. From a methodological perspective, these tables provide a structured overview of the technological ecosystems and data acquisition strategies that underpin the development of such systems in the current literature. Figure 6 shows these basic taxonomies.

Table 3a–c classify the reviewed articles according to the technological functions used in implementing DTs. The taxonomy groups technologies into functional categories—such as spatial capture, sensing and monitoring, geospatial analysis, immersive visualization, artificial intelligence, and DT infrastructures—thereby highlighting the technological layers that typically compose DT architectures in tourism and hospitality. This functional classification enables the identification of prevailing technological trends, and the relative emphasis on data acquisition, modelling, analytics, and user interaction across the literature.

Complementarily, Table 4a,b organize the same body of articles according to their primary data sources. This perspective focuses on the types of information used to feed and update DTs, including environmental sensing data, spatial datasets, user behavioral data, social media content, and documentary or institutional sources. Analyzing the literature through the lens of data provenance helps clarify how SDTs integrate heterogeneous datasets to represent both the physical and social dimensions of tourism environments.

Building upon the technological and data patterns identified in Table 3 and Table 4, the analysis highlights that current DT implementations in tourism and hospitality rely on increasingly heterogeneous sensing technologies capable of capturing spatial, physiological, and behavioral dimensions of service environments. As reflected in the reviewed studies, technologies such as indoor localization, wearable sensing, and computer vision enable the real-time observation of guest flows, staff movement, physiological conditions, and service interactions, generating complementary data streams that describe both operational processes and human activity within hospitality contexts [4,7]. This growing technological complexity underscores the need for approaches that can integrate and interpret heterogeneous data sources within a unified representation of the system [4]. In this context, semantic and ontology-driven technologies play a critical role by providing structured knowledge models that allow the integration of multiple data streams and the explicit representation of relationships between physical infrastructures, digital systems, operational processes, and human actors [12,13]. Similar challenges related to data integration, system coordination, and real-time decision support have also been addressed in other DT application domains, where recent studies explore the use of digital replicas to support dynamic system management and optimization in complex environments [91,92,93,94].

By enabling this semantic layer, DTs can move beyond fragmented data-driven representations and support a more comprehensive understanding of the socio-technical complexity that characterizes tourism and hospitality environments and dynamics. Overall, this analysis facilitates the identification of dominant methodological patterns and emerging research directions in the application of SDTs to tourism contexts.

### 7.3. Relational Analysis Between Technology Stack, Semantic Design, and Human Integration

The comparative taxonomy presented in the previous section highlights the structural diversity of DT implementations in hospitality. However, beyond classification, it is necessary to examine how key dimensions interact and co-evolve, particularly the relationships between technological configurations, semantic architectures, and the degree of human integration. This analysis addresses that gap by identifying cross-dimensional correlations observed both in the reviewed literature and in the comparative analysis of the reviewed studies [15].

A consistent pattern across the literature is that increased technological complexity does not directly translate into higher levels of human integration. Most studies employ heterogeneous sensing technologies, including IoT infrastructures, computer vision, and geospatial systems, yet continue to represent humans primarily as system users or sensed entities rather than as core model components [64,71]. This reveals a structural decoupling between data acquisition and human-centric modeling. While sensing technologies increase system observability, human integration depends on the system’s ability to semantically interpret, reason about, and act upon human-related data. Evidence from the proposed framework reinforces this distinction. Although IoT and real-time data streams enable situational awareness, improvements in human-centric outcomes, such as satisfaction and contextual adaptation, only emerge when reasoning and personalization layers are incorporated. This suggests that human integration is not driven by sensing capabilities alone, but by higher-level semantic and cognitive processes.

A second correlation concerns the role of ontology-driven architectures in enabling reasoning capabilities. Although relatively limited in number, ontology-based DTs in the literature are consistently associated with semantic interoperability, structured knowledge representation, and context-aware inference. Ontologies therefore function not only as integration mechanisms, but as foundational enablers of reasoning. By structuring entities and their relationships within a formal semantic model, they allow the system to infer context, interpret interactions, and support decision-making processes grounded in human behavior. This capability is particularly relevant in complex service environments, where outcomes depend on dynamic interactions between actors, processes, and contextual conditions. This aligns with prior work identifying ontologies as a key enabler of semantic reasoning and knowledge inference in DT systems [12,14].

The interaction between reasoning and personalization further clarifies how human-centric outcomes emerge. Evidence across the literature indicates that both components independently improve system performance, but their combined effect is significantly greater. Configurations incorporating reasoning improve operational efficiency and contextual alignment, while those including personalization enhance user satisfaction. However, the literature suggests that the most advanced configurations are those in which ontology, reasoning, and personalization are integrated. This indicates a non-linear relationship between system components. Human-centricity does not arise from any single capability, but from the interaction between semantic understanding, reasoning processes, and personalization mechanisms. Systems lacking one of these elements tend to remain either operationally efficient but impersonal, or personalized but contextually limited. This observation is consistent with human-centric AI perspectives emphasizing the integration of reasoning and personalization in adaptive systems [22,23].

A similar pattern is observed in relation to data heterogeneity. The literature consistently highlights the diversity of data sources used in DT systems, including IoT telemetry, behavioral data, and user-generated content. However, the analysis shows that data diversity alone does not guarantee performance improvements. Gains are observed only when heterogeneous data is semantically integrated. Without a semantic layer, data remains fragmented, limiting the system’s ability to establish relationships and perform cross-source reasoning. In contrast, ontology-driven integration transforms disparate data streams into contextualized knowledge, enabling the system to infer relationships across domains, such as linking guest preferences, staff workload, and environmental conditions. This confirms that semantic integration is a prerequisite for effective reasoning, interoperability, and explainability in DT systems. This is consistent with prior research identifying semantic integration as a prerequisite for interoperability and cross-domain reasoning [4,12,21].

**Figure 7:**
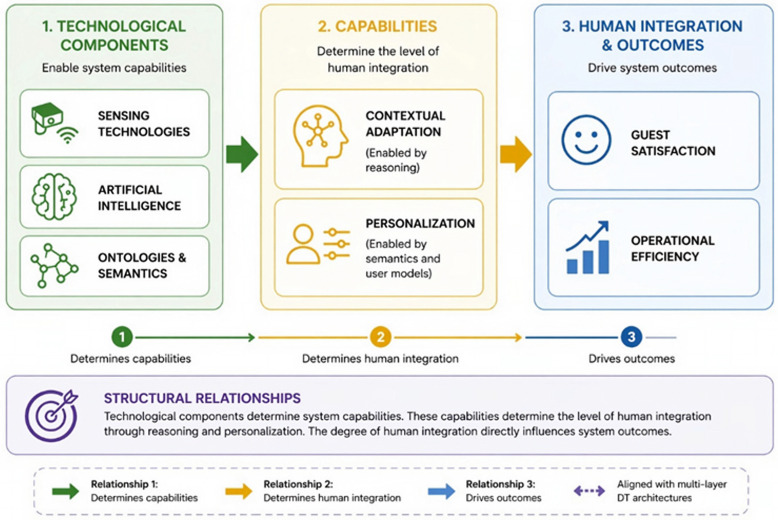
Structural relationships between technological components, system capabilities, human integration, and outcomes in human-centric DT. This figure illustrates the conceptual relationships underlying human-centric DTs. Technological components, including sensing technologies, artificial intelligence, and semantic models, determine system capabilities such as observability, prediction, and reasoning. These capabilities influence the level of human integration through contextual adaptation and personalization mechanisms. In turn, human integration drives system outcomes, including improved guest satisfaction, reduced operational delays, and increased staff efficiency. The figure highlights that system performance depends not only on technological sophistication but on the architectural and semantic integration of these components.

Taken together, these observations reveal three structural relationships. First, technological components determine system capabilities: sensing technologies increase observability, artificial intelligence enables prediction and optimization, and ontologies enable reasoning and semantic interpretation. Second, these capabilities determine the level of human integration, as reasoning enables contextual adaptation and personalization enables user-specific responses. Third, the degree of human integration directly influences system outcomes, including improvements in guest satisfaction, reductions in operational delays, and increased staff efficiency. These relationships indicate that human-centric DTs are not simply the result of technological sophistication, but of how technologies are architecturally combined and semantically structured. These relationships align with prior work describing DT systems as multi-layered architectures where data acquisition, modeling, and decision-making capabilities evolve jointly [4,7]. Figure 7 illustrates these structural relationships.

Despite these advantages, several challenges remain. Reasoning over large semantic graphs introduces computational overhead, which can affect real-time performance. Increased human integration also raises privacy and ethical concerns, particularly when behavioral and physiological data are involved. In addition, integrating heterogeneous data sources requires robust semantic alignment mechanisms, increasing system complexity. These challenges highlight the need for balanced architectures that carefully manage trade-offs between performance, interpretability, and ethical constraints. These challenges are widely documented in DT and AI systems, particularly in relation to scalability, privacy, and ethical data use [15,16,27,42,43].

Overall, the analysis demonstrates that ontology-driven approaches are consistently associated with higher reasoning capability and improved contextual accuracy, while human integration depends primarily on semantic and cognitive layers rather than on sensing technologies alone. The highest levels of system performance are achieved when ontology, reasoning, and personalization are combined. This shifts the perspective on DTs from purely data-intensive systems toward semantically structured socio-technical systems, where human-centric outcomes emerge from the interaction between technological components and conceptual design choices [15,16].

These relationships are derived from cross-comparison of the reviewed studies and should be interpreted as indicative patterns rather than causal relationships.

### 7.4. Key Research Gaps

The comparative analysis presented in the previous section provides an overview of how DT research in tourism and hospitality is currently structured across the proposed taxonomy. Beyond describing the distribution of studies across different application levels, modelling focuses, technological architectures, and forms of human integration, the review also highlights several limitations and underexplored areas within the existing body of literature. These observations identify key research gaps that remain inadequately addressed in current studies and represent important directions for future research. In addition to the patterns observed in the comparative taxonomy, the review also identifies limitations in the conceptualization, implementation, and governance of DTs in tourism contexts.

Low level of governance and socio-technical integration. One of the most evident gaps in the reviewed literature concerns the limited attention to governance and socio-technical design aspects in the development of DTs for tourism and hospitality. While most studies focus primarily on technological infrastructures, data architectures, or system functionalities, governance mechanisms and socio-technical dynamics are rarely addressed. The analysis of the reviewed studies shows that 30 of the 42 analyzed contributions do not explicitly incorporate governance structures, stakeholder coordination mechanisms, or organizational decision-making processes within the proposed DT frameworks. These findings suggest that many DT initiatives in tourism are conceptualized mainly as technological systems rather than as socio-technical systems embedded within complex tourism ecosystems. However, tourism environments typically involve multiple stakeholders, institutional arrangements, and interdependent processes [95,96], making governance considerations essential for the effective implementation, coordination, and long-term sustainability of DT solutions.

Conceptual ambiguity in the use of the Digital Twin concept. Another important gap identified in the reviewed literature concerns the conceptual ambiguity surrounding the use of the DT concept in tourism and hospitality research. Although many studies use the term DT to describe their proposed systems, the technological characteristics of fully developed DTs are not always clearly implemented. According to widely accepted definitions, a fundamental feature of DTs is the continuous exchange of data between the physical system and its digital counterpart, which enables the digital representation to dynamically reflect the state of the real-world system [2,3,4]. However, this characteristic is frequently absent or only partially addressed in the reviewed studies. The analysis shows that 19 of the contributions do not incorporate real-time synchronization mechanisms, 14 include only partial synchronization, and only 9 explicitly consider continuous real-time data exchange between the physical and digital systems. These findings suggest that, in many cases, the proposed solutions align more closely with digital models, simulation environments, or data-driven representations than with fully operational DTs. This observation indicates that the term DT is sometimes used in a broad, loosely defined manner in the tourism and hospitality literature.

Limited development of human-centered Digital Twins. Another important gap identified in the reviewed literature concerns the limited development of human-centered DTs in tourism and hospitality contexts. The comparative analysis shows that none of the studies analyzed focus on DTs at the individual level, indicating that individual actors, such as tourists or employees, are rarely treated as the primary entities represented by the digital model. Similarly, only two of the reviewed studies adopt a human-centric modelling perspective, and these are the same contributions that position the human element as the core model entity within the DT. In most cases, humans are incorporated only indirectly, typically as system users interacting with the DT or as sensed entities whose data are used as inputs for system monitoring and analysis. This suggests that the majority of DT applications in tourism remain oriented towards environments, infrastructures, or operational systems rather than towards the representation of human actors and their behaviors. Considering that tourism systems are fundamentally human-centered and involve complex interactions between tourists, employees, and service environments [8,9], the limited development of human-centered DTs represents a significant gap in the current literature and highlights an important direction for future research.

**Figure 8:**
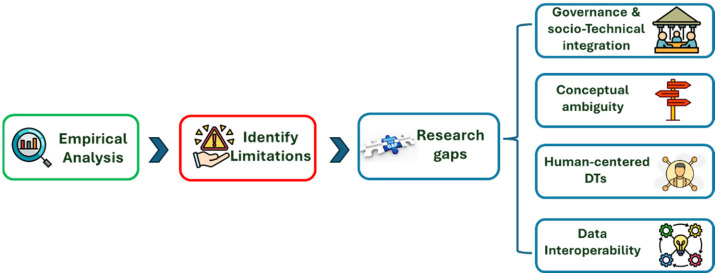
From empirical patterns to research gaps in Digital Twin research in tourism and hospitality. The figure illustrates the progression from the empirical analysis of the reviewed studies to the identification of key limitations and the definition of research gaps, including governance and socio-technical integration, conceptual ambiguity, human-centered DTs, and data interoperability.

Data interoperability and integration challenges. The technological and data-related analysis presented in Table 3 and Table 4 also reveals important challenges related to data interoperability and integration within DT systems. The reviewed studies rely on highly heterogeneous data sources, including IoT sensors, geospatial datasets, behavioral tracking data, social media information, and expert or documentary sources. While these diverse data streams illustrate the richness of potential input sources for DT models, the literature rarely discusses how heterogeneous datasets can be effectively integrated into unified DT architectures. Issues related to data interoperability, standardization, and cross-platform integration, therefore, remain largely unexplored in tourism-oriented DT research. Figure 8 provides an integrative overview linking empirical findings with identified limitations and the resulting research gaps, highlighting the main directions for future research.

## 8. Conclusions and Future Research Directions

This study provides a systematic analysis of the current state of DT research in tourism and hospitality, offering a structured understanding of how these systems are conceptualized, implemented, and integrated within service-oriented environments. By combining a comparative taxonomy with a technological and data-oriented analysis, the review not only identifies dominant patterns in the literature but also reveals critical limitations that shape future research directions.

The findings highlight a clear misalignment between the inherently human-centered nature of tourism and hospitality systems and the way DTs are currently designed. Despite the central role of human interactions, relationships, and service encounters in these contexts, most DT applications are developed at the destination and facility levels, with no evidence of individual-level DTs. Similarly, although human actors are frequently incorporated into DT systems, they are typically positioned as system users or data sources rather than as core modelling entities. This limits the capacity of current DT implementations to capture the complexity, dynamism, and socio-technical nature of tourism service systems.

In addition, the analysis reveals that governance and socio-technical dimensions remain largely underexplored. Most studies focus on technological infrastructures and data-driven functionalities, while aspects such as stakeholder coordination, institutional frameworks, and regulatory considerations are rarely addressed. This reinforces the tendency to conceptualize DTs as purely technological artefacts rather than as socio-technical systems embedded within complex tourism ecosystems.

The review also identifies key challenges in conceptualizing DTs. A significant proportion of the analyzed studies do not fully implement a key characteristic of DT systems: real-time synchronization between physical and digital entities. With only a limited number of studies incorporating continuous data exchange, many proposed solutions resemble data-driven models or simulation environments rather than fully operational DTs. In addition to the presence of studies that loosely adopt the DT terminology, these findings point to a lack of conceptual rigor in the application of the Digital Twin concept in tourism and hospitality research.

From a technological perspective, the literature reveals a significant heterogeneity across sensing technologies and data sources, including IoT infrastructures, geospatial systems, behavioral tracking data, and immersive technologies. While this diversity reflects the richness of potential input sources for DT systems, it also introduces significant challenges in data integration and interoperability. In this context, the findings highlight the importance of semantic and ontology-driven approaches as key enablers for integrating heterogeneous data sources and representing the complex relationships between technologies, processes, and human actors within tourism environments.

Building on these findings, this study advocates a shift towards SDT ecosystems in tourism and hospitality. Such systems extend traditional DT architectures by integrating not only physical infrastructures and operational processes but also human actors, social interactions, and governance structures within a unified modelling framework. This perspective emphasizes the need to move beyond technology-centric approaches towards socio-technical systems capable of capturing the complexity of service environments. However, achieving this transition involves addressing several interrelated challenges. From a technical perspective, issues related to data interoperability, real-time processing, and system scalability remain critical, particularly in data-intensive environments. From a human-centric perspective, the development of SDTs requires advancing beyond the current limited representation of human actors to incorporate behavioral dynamics, service interactions, and context-dependent decision-making processes. An additional critical implication concerns the integration of human-related data and its associated ethical and organizational challenges. As DT systems increasingly incorporate information derived from human behavior, physiological sensing, and interaction patterns, issues related to privacy, trust, and user acceptance become central to their successful deployment. Organizations must therefore balance the benefits of data-driven optimization—such as improved service performance and operational efficiency—with the need to protect the privacy and autonomy of employees, customers, and other stakeholders. Without transparent data governance practices and clear communication about data use, sensing-driven DT systems may foster perceptions of surveillance and reduce user acceptance. Consequently, the development of SDTs requires trust-based frameworks that ensure transparency, data protection, and user control, as well as further research into the conditions under which individuals are willing to adopt and interact with such systems. In this context, an additional challenge emerges from the tension between the need for high-fidelity data to model complex human behavior and the principles of data minimization and privacy-by-design established by regulatory frameworks such as the GDPR. While SDTs rely on rich, continuous, and context-sensitive data to accurately represent human interactions and decision-making processes, these requirements may conflict with constraints on data collection and processing. Addressing this trade-off represents a critical direction for future research, requiring approaches that balance model accuracy with privacy protection, transparency, and user control. From a policy and regulatory perspective, these challenges highlight the need for clear governance frameworks to guide the development and deployment of SDTs in tourism and hospitality. In particular, ensuring compliance with data protection regulations such as GDPR, while enabling innovation in data-driven modelling, requires coordinated efforts between policymakers, industry stakeholders, and technology developers. Establishing transparent and accountable regulatory frameworks will be essential to support responsible adoption and foster trust in these systems.

Taken together, these findings not only highlight the limitations of current Digital Twin implementations but also provide a foundation for advancing the field. In this regard, this study contributes to the literature in several ways. It proposes a structured taxonomy that enables a systematic comparison of DT applications in tourism and hospitality, and it identifies key limitations in current DT implementations, particularly regarding human integration, governance, and conceptual clarity. Moreover, it advances the discussion towards SDTs by highlighting the importance of semantic integration and socio-technical modelling approaches as fundamental components of future DT systems. From a computational perspective, the integration of high-velocity and heterogeneous data streams poses significant challenges for ontology-driven SDTs, particularly in relation to the scalability of semantic reasoning processes. In this context, recent approaches [15,16] suggest the need for hybrid architectural strategies in which real-time data processing and semantic reasoning are partially decoupled. Under this perspective, semantic inference can be selectively activated in response to relevant events, allowing ontology-driven models to provide contextual interpretation and decision support without compromising system responsiveness. This highlights the importance of moving from monolithic semantic architectures toward more modular and adaptive designs that balance analytical performance with explainability. Further research is needed to explore and validate these approaches in real-world hospitality environments. From a practical perspective, the findings provide relevant insights for organizations and destination managers seeking to implement DT technologies. The results suggest that current DT approaches may be insufficient if they do not adequately incorporate human and organizational dimensions. The adoption of semantic and ontology-driven architecture, together with human-centric data integration, can support the development of more comprehensive, context-aware systems that improve decision-making and service management in tourism environments.

While these contributions offer relevant theoretical and practical insights, the results of this study should be interpreted with certain limitations in mind. The review is restricted to publications indexed in Scopus and Web of Science, potentially excluding relevant contributions from other sources. Additionally, reliance on these databases may introduce a disciplinary bias, as they are predominantly oriented towards STEM-related publications. This may lead to an overrepresentation of technically focused studies and a potential underrepresentation of conceptual, managerial, or socio-organizational contributions more commonly found in tourism and social science outlets. Furthermore, the review may be subject to publication bias, as it is based on peer-reviewed academic literature. In this context, certain types of contributions (such as preliminary results, unsuccessful implementations, or non-published industry practices) may be underrepresented. As a result, the reviewed studies may not fully capture the entire range of experiences and developments related to DT applications in tourism and hospitality. In addition, only studies published in English were considered, which may have led to the omission of relevant research available in other languages. Finally, although a systematic methodology was applied, the classification of studies involves some interpretative judgment. In addition, the proposed taxonomy is intended to describe, structure, and synthesize the current state of DT research in tourism and hospitality, while supporting a systematic and comparative analysis of existing applications. Future research could extend this framework by incorporating measurable indicators, maturity levels, and explanatory factors that enable a more systematic assessment of DT systems and provide deeper analytical insights. Moreover, research efforts should also explore the development of human-centered, socially aware DT architectures, particularly at the individual level, as well as the integration of governance mechanisms and interoperable semantic frameworks to support scalable, context-aware SDT ecosystems.

Building on these directions, future research would benefit from more operationalized and empirically grounded approaches. In particular, several research questions emerge from the findings of this study: (i) how can the maturity of DT systems be systematically assessed, particularly in relation to human integration and socio-technical complexity?, (ii) what types of semantic models and ontologies are required to represent social interactions, roles, and organizational dynamics in tourism and hospitality contexts?, (iii) how can real-time synchronization mechanisms be effectively implemented and evaluated in complex service environments?, (iv) what governance frameworks and organizational structures are needed to support the deployment of SDTs?, and (v) under what conditions are employees and users willing to adopt and interact with systems that integrate behavioral and physiological data?

Addressing these questions would contribute to bridging the gap between current technological approaches and the development of more comprehensive, human-centered Digital Twin ecosystems.

Ultimately, advancing towards SDT ecosystems represents not only a technological evolution but a paradigm shift towards more human-centered, integrated, and context-aware representations of tourism systems, opening new avenues for research and practice in the digital transformation of the sector.

## Figures and Tables

**Table 1:** (**a**). Comparative classification of DT studies in tourism and hospitality according to four dimensions: application level, twin focus, architectural approach, and human integration (Destination-level). (**b**). Comparative classification of DT studies in tourism and hospitality according to four dimensions: application level, twin focus, architectural approach, and human integration (Facility-level). (**c**). Comparative classification of DT studies in tourism and hospitality according to four dimensions: application level, twin focus, architectural approach, and human integration (Process-level).

(a)			
Study	Twin Focus	Arch. Approach	Human Integration
[33,34,35,36,59,60,61]	Environment-centric	Data-driven	System users
[37]	Environment-centric	Hybrid	Sensed entities
[62]	Process-centric	Hybrid	System users
[63,64]	Environment-centric	Data-driven	System users + sensed entities
[65]	Process-centric	Hybrid	System users + sensed entities
[39,66]	Environment-centric	Hybrid	System users + sensed entities
[67]	Environment-centric	Data-driven	None
[38]	Environment-centric	Data-driven	Sensed entities
[68]	Process-centric	Simulation-based	None
[69,70]	Environment-centric	None	System users
[71]	Hybrid	None	System users
**(b)**			
**Study**	**Twin Focus**	**Arch. Approach**	**Human Integration**
[72]	Asset-centric	Hybrid	None
[15,16]	Human-centric	Ontology-driven	System users + sensed entities + core model entity
[73]	Asset-centric	Data-driven	None
[74]	Asset-centric	Hybrid	None
[75,76]	Asset-centric	Simulation-based	Sensed entities
[77,78,79]	Asset-centric	Data-driven	System users
[80,81]	Hybrid	Hybrid	System users + sensed entities
[82]	Process-centric	None	System users
[41]	Hybrid	Ontology-driven	System users + sensed entities
[83]	Hybrid	Hybrid	System users
[84]	Process-centric	Data-driven	None
[85]	Asset-centric	Simulation-based	System users
[40]	Asset-centric	AI-enhanced	System users + sensed entities
[86]	Environment-centric	Data-driven	System users
[87]	Asset-centric	AI-enhanced	System users
**(c)**			
**Study**	**Twin Focus**	**Arch. Approach**	**Human Integration**
[88]	Environment-centric	Data-driven	System users + sensed entities
[89]	Environment-centric	AI-enhanced	System users

**Table 2:** Distribution of reviewed studies according to their level of real-time synchronization. This classification provides additional insight into the extent to which the analyzed systems meet one of the defining characteristics of DTs, namely continuous data exchange between physical and digital entities.

Real-Time Synchronization	Number of Studies	References
Yes	9	[15,16,33,38,39,65,72,74,80]
Partial	14	[35,40,41,62,64,66,70,75,76,81,86,87,88,89]
No	19	[34,36,37,59,60,61,63,67,68,69,71,73,77,79,82,83,84,85,90]

**Table 3:** (**a**): Technologies for analysis, exploitation, and interaction, based on user experience. (**b**)**:** Technologies for capture and acquisition of the physical world, oriented towards measuring, observing, and digitizing reality. (**c**): Technologies for modeling, representation, and semantic data management, used for the creation of digital representations.

(a)				
Technologies	Description	Function	Solution	Papers
AI & Intelligent Analytics	Technologies used to analyze data and support automated decision-making.	Machine Learning Methods	Supervised, Deep and Federated learning	[65]
		Computer Vision Techniques	Object detection (YOLO), Image recognition, Scene understanding	[37]
		Predictive and Optimization Models	Reinforcement learning, Predictive analytics, Decision support systems	[68,69,71,80]
		Generative and Cognitive AI	Large Language Models (LLMs), Generative AI models, AI knowledge assistants	[80]
Immersive Visualization & Interaction	Technologies enabling interactive exploration of digital environments.	Virtual Reality Systems	VR headsets, VR simulation environments, VR tourism platforms	[61,85,89]
		Augmented and Mixed Reality	AR mobile applications, HoloLens systems, Mixed reality visualization	[39,40]
		Extended Reality Platforms	XR Interaction Toolkit, collaborative platforms and visualization environments	[36]
		Metaverse and Virtual Worlds	Metaverse platforms, Virtual city environments, Shared virtual tourism spaces	[34,66,86]
**(b)**				
**Technologies**	**Description**	**Function**	**Solution**	**Papers**
Sensing & Environmental Monitoring	Technologies that collect dynamic data from the environment	Environmental Monitoring Sensors	Environmental sensors	[84,88]
		Infrastructure Monitoring Systems	Energy monitoring systems, Smart building telemetry, Structural monitoring sensors	[72,75]
		Urban IoT Sensing	IoT sensor, Smart infrastructure sensing, City monitoring systems	[64]
		Spatial Sensing Technologies	LiDAR sensing, Depth sensors, Proximity sensors	[16,38]
Geospatial & Location Technologies	Technologies used to analyze and manage spatial information	Geographic Information Systems	GIS platforms, Spatial data analysis tools, Geospatial databases	[35,67]
		Location-Based Services	GPS positioning systems, Location-aware applications, Mobile geolocation services	[16]
		Spatial Data Infrastructure	OpenStreetMap APIs, National spatial data infrastructures, Geospatial web services	[67]
		Spatial Analytics	Geospatial analysis, Spatial data mining, Spatial simulation models	[62]
Spatial Capture & 3D Digitization	Technologies used to capture geometric or visual data from the physical world	Laser-Based Spatial Scanning	LiDAR scanning, Terrestrial laser scanning, Mobile LiDAR scanning	[73,79]
		Image-Based 3D Capture	Photogrammetry / Structure-from-Motion (SfM), Smartphone image capture	[33,59,77,90]
		Hybrid Spatial Acquisition	PhoDAR systems, SLAM mobile scanning, Multi-sensor mapping systems	[60]
		Panoramic and Immersive Imaging	Panoramic cameras, 360° imaging systems, Mobile panoramic capture	[63]
**(c)**				
**Technologies**	**Description**	**Function**	**Solution**	**Papers**
3D Modelling & Digital Representation	Technologies used to generate structured digital models from captured data	3D Reconstruction Techniques	Mesh reconstruction, Point cloud processing, Surface reconstruction	[41,81]
		Built Environment Modelling	BIM & HBIM Modelling	[74,76]
		Urban and Territorial Models	3D city models, Digital environment Modelling, Urban digital twins	[66]
		Semantic 3D Representation	Semantic 3D Modelling, Ontology-linked models, Structured digital environments	[82]
Semantic Web & Knowledge Modelling	Technologies used to represent knowledge and relationships between entities.	Semantic Data Standards	RDF, OWL, Linked data	[15]
		Query and Reasoning Systems	SPARQL query systems, Semantic reasoning engines, Ontology-based inference	[15]
		Knowledge Representation Systems	Knowledge graphs, Semantic databases, Ontology-based DTs	[82]
		Semantic Integration	Linked data integration, Semantic interoperability frameworks, Semantic digital twin architectures	[15,82]
Digital Twin Platforms & Data Infrastructure	Technologies supporting data integration and system orchestration	Digital Twin Platforms	Commercial DT platforms	[70]
		Data Integration Frameworks	Data fusion systems, Multi-source data integration, Data pipelines	[83]
		Real-Time Data Infrastructure	Real-time synchronization systems, Streaming architectures, Edge-cloud data architectures	[87]
		Digital Twin Architectures	Twin orchestration systems, Multi-layer twin architectures, Simulation-driven twin platforms	[70,83]

**Table 4:** (**a**): Instrumented and observational data, acquired through direct measurement of the physical environment via sensors and positioning systems. (**b**): Human-generated and behavioral data, arising from human activity, interactions, and digital footprints.

(a)				
Data Origin Domain	Description	Data Acquisition System	Example Data Types	Papers
Physical Environment Data	Data describing the geometry, structure, or physical condition of the environment.	3D Geometric Capture	Laser scanning point clouds, LiDAR scans, UAV photogrammetry, SLAM mapping, 3D mesh reconstructions	[33,37,38,59,60,61,66,73,74,79,81,89,90]
		Environmental Imagery and Video	Photogrammetric, Panoramic and UAV imagery, Surveillance camera footage, multi-camera datasets	[16,33,37,39,60,61,63,64,65,73,74,77,85,90]
		Existing Digital Models	BIM, HBIM, CAD and 3D city models	[33,36,37,38,40,59,60,62,85]
Sensor & IoT Monitoring Data	Data generated by sensors monitoring dynamic system conditions	Environmental Sensors	Temperature, humidity or CO_2_ measurements, Noise levels, Air quality monitoring	[15,33,39,64,74,75,76,80]
		Infrastructure Sensors	HVAC performance, Energy consumption, Smart building telemetry	[15,72,76,80,84]
		Urban Sensors	Traffic, Smart mobility & Infrastructure monitoring sensors	[33,35,64]
Geospatial Data	Structured data describing location and spatial organization	Digital Cartography	OpenStreetMap datasets, GIS layers, Municipal spatial datasets	[33,35,37,59,60,62,66,67,74]
		Tourism Infrastructure Data	Points-of-interest (POI) datasets, Tourism infrastructure databases	[15,33,37,38,62,67,73,74,76,89]
		Official Territorial Data	Government geospatial datasets, Urban planning datasets	[62,64]
**(b)**				
**Data Origin Domain**	**Description**	**Data Acquisition System**	**Example Data Types**	**Papers**
Human Behavior Data	Data describing human activity, movement, or interaction	Motion and Posture Tracking	Gesture datasets, Body posture tracking, Motion capture data	[16,34,39,63,64,65,80,88,89]
		Mobility and Trajectories	Visitor trajectory logs, Pedestrian tracking data, Mobility traces	[37,38,40,62,63,75,79,81]
		Digital Interaction	XR interaction logs, User interaction telemetry	[36,40,41,81]
Social & Digital Platform Data	Data generated through online platforms and digital services	Social Media Data	Geotagged social media posts, Social media images and text data	[37,41,64]
		User-Generated Content	Online reviews, Check-ins, Travel logs	[15,16,36,41,59,63,64,69,74,79]

## Data Availability

Data is contained within the article.

## Abbreviations

The following abbreviations are used in this manuscript:

DT	Digital Twin
SDT	Social Digital Twin
WoS	Web of Science
